# Whole Grain Intake and Glycaemic Control in Healthy Subjects: A Systematic Review and Meta-Analysis of Randomized Controlled Trials

**DOI:** 10.3390/nu9070769

**Published:** 2017-07-19

**Authors:** Stefano Marventano, Claudia Vetrani, Marilena Vitale, Justyna Godos, Gabriele Riccardi, Giuseppe Grosso

**Affiliations:** 1 Department of Medical and Surgical Sciences and Advanced Technologies “G.F. Ingrassia”, University of Catania, 95123 Catania, Italy; stefano.marventano@studium.unict.it; 2 Department of Clinical Medicine and Surgery, “Federico II” University, 80131 Naples, Italy; c.vetrani@libero.it (C.V.); riccardi@unina.it (G.R.); 3 Integrated Cancer Registry of Catania-Messina-Siracusa-Enna, Azienda Ospedaliero-Universitaria Policlinico Vittorio Emanuele, 95124 Catania, Italy; justyna.godos@student.uj.edu.pl (J.G.); giuseppe.grosso@studium.unict.it (G.G.)

**Keywords:** whole grain, glycemia, insulin, healthy subjects, meta-analysis, RTC

## Abstract

Backgrounds: There is growing evidence from both observational and intervention studies that Whole Grain (WG) cereals exert beneficial effects on human health, especially on the metabolic profile. The aim of this study was to perform a meta-analysis of randomised controlled trials (RCT) to assess the acute and medium/long-term effect of WG foods on glycaemic control and insulin sensitivity in healthy individuals. Methods: A search for all the published RCT on the effect of WG food intake on glycaemic and insulin response was performed up to December 2016. Effect size consisted of mean difference (MD) and 95% CI between the outcomes of intervention and the control groups using the generic inverse-variance random effects model. Results: The meta-analysis of the 14 studies testing the acute effects of WG foods showed significant reductions of the post-prandial values of the glucose iAUC (0–120 min) by −29.71 mmol min/L (95% CI: −43.57, −15.85 mmol min/L), the insulin iAUC (0–120 min) by −2.01 nmol min/L (95% CI: −2.88, −1.14 nmol min/L), and the maximal glucose and insulin response. In 16 medium- and long-term RCTs, effects of WG foods on fasting glucose and insulin and homeostatic model assessment-insulin resistance values were not significant. Conclusions: The consumption of WG foods is able to improve acutely the postprandial glucose and insulin homeostasis compared to similar refined foods in healthy subjects. Further research is needed to better understand the long-term effects and the biological mechanisms.

## 1. Introduction 

Over the past decades, the burden of non-communicable chronic diseases, such as cardiovascular diseases (CVD), metabolic syndrome, diabetes and cancer, is rapidly increasing worldwide [1]. Non-communicable chronic diseases are generally preventable by managing modifiable risks factors, including dietary habits. Diet may play a key role in the promotion and maintenance of good health, but modern lifestyle is leading to a shifting away from traditional dietary patterns in favour of eating habits characterized by increased portion sizes, away-from-home foods, and unhealthy snacking. This process, known also as “Westernization” of the diet, is characterized by major consumption of refined sugars and fats, animal products and refined cereals, as well as decreased consumption of fruit, vegetable, and whole-grain (WG) cereals. 

WG cereals contain all three parts of a natural grain kernel, including endosperm, germ, and bran and they are a rich source of dietary fibre, resistant starch, antioxidants, vitamins and minerals, such as folic acid, magnesium, and selenium [2]. The refinement process is performed by removing the bran and some of the germ, resulting in a loss of micronutrients and dietary fibre in favour of a softer texture and extended freshness. Thus, the endosperm alone, mainly composed of starchy carbohydrates, proteins, and small amounts of vitamins and minerals, is used to produce refined white flours.

There is growing evidence from both observational and intervention studies that WG cereals exert beneficial effects on human health [3]. WG food consumption has been associated with a modification of risk factors related to non-communicable chronic diseases, including post-prandial insulinaemic response, blood lipid profile, and intestinal microbiota [4,5,6]. A series of previous meta-analyses have shown a decreased risk of CVD [7], type-2 diabetes [8], metabolic syndrome [9], and cancer [10] associated with higher intake of WG foods. The mechanisms underlying the health benefits of WG foods may be explained, at least in part, by the high content of fibre that positively affects gut health (increasing transit time and faecal bulking). Moreover, other mechanisms, such as anti-inflammatory, antioxidant, hormonal (i.e., in hormone activation and synthesis of zinc, selenium, and nicotinic acid), anti-carcinogenic (i.e., content in phenolic compounds), and metabolic effects (i.e., vitamins and minerals), have to be considered [11].

A previous meta-analysis of 14 cohorts showed that WG food consumption was associated with lower fasting glucose and insulin concentrations independently from demographics and lifestyle factors, in non-diabetic European descents [12]. Although several clinical trials have been carried out to show the effects of WG consumption in healthy individuals, results are still conflicting. The aim of this study was to perform a meta-analysis of randomised controlled trials (RCT) to assess the acute and medium/long-term effect of WG foods on glycaemic control and insulin sensitivity in healthy individuals.

## 2. Methods

### 2.1. Search Strategy and Selection of the Studies

A search for all the published RCT on the effect of WG intake on glycaemic and insulin response was performed independently by three authors (J.G., C.V. and M.V.). Studies were identified from PubMed, Science Direct Online and The Cochrane Library up to December 2016. The search strategy included following key words: (“whole grain” or” whole meal” or “whole wheat” or “whole kernel”) and (“blood glucose” or “HbAlc” or “glycated haemoglobin” or “fasting plasma glucose” or “glycolated haemoglobin” or “FBG” or “insulin” or “HOMA-IR”). The search was restricted to studies published in English. The reference list of the included studies was checked to identify relevant study not previously included. Inclusion criteria were: (i) studies conducted on healthy human subjects; (ii) parallel or crossover design; (iii) comparison of WG foods with foods with lower levels or no WG content; and (iv) presented means, standard deviations (SDs), or standards errors (SEs) at baseline and/or endpoint for the outcomes investigated. Exclusion criteria were the following: (i) studies with different design; (ii) studies evaluated only individual components of the grain; (iii) studies in which it was not possible to evaluate the WG foods effect because part of combined meals; and (iv) studies with fewer than 10 subjects in the intervention group.

### 2.2. Data Extraction and Study Quality

Data were independently extracted by three reviewers using a standard form. The information extracted included: first author and year of publication, country, study design, participant characteristics, number of subjects in comparison groups, type of WG foods, length of intervention, matching characteristics, and main results of the outcomes investigated. Primary outcomes consisted of changes from baseline in fasting glucose and insulin concentrations and in glucose and insulin incremental area under the curve (iAUC) values. Secondary outcomes included mean changes in HbA1c concentrations, peak and incremental glucose and insulin and homeostatic model assessment-insulin resistance (HOMA-IR) values. Changes from baseline were chosen as primary outcome as in many studies they were the only data presented, although this might not be the best approach (Bland and Altman). 

The quality of each study was assessed following the principles of the Newcastle-Ottawa Quality Assessment Scale [13], consisting of 3 domains of quality as follows: selection (4 points), comparability (2 points), and outcome (3 points) for a total score of 9 points (9 representing the highest quality). Studies scoring 0–3 points, 3–6 points, and 7–9 points were identified as low, moderate, and high quality, respectively.

### 2.3. Statistical Analysis

In order to perform the comparison, all the values were converted to mmol/L for glucose concentrations and pmol/L for insulin concentrations. IAUC were converted to min x mmol/L for glucose iAUC values and min x nmol/L for insulin iAUC. When insulin was reported in μIU/mL (microunits of insulin per millilitre), a conversion factor of 6.945 was applied to convert to pmol/L (picomoles of insulin per litre) [14]. Change from baseline to endpoint was used for the analysis of fasting glucose, fasting insulin and HOMA-IR. When SD was not reported, it was derived from available data (95% confidence interval (CI), *p*-values, t or F statistics, and SE) using the method suggested by the Handbook for Systematic Review of Interventions [15]. When needed, the SD for changes from baseline was imputed using a pooled correlation coefficient according to a published procedure [15]. When it was not possible, a correlation coefficient of 0.5 was applied for imputing missing SDs, as it is a conservative value between 0 and 1. End-of-treatment values were used for glucose and insulin iAUC while data on HbA1 were insufficient to perform a meta-analysis. Results of trials containing multiple intervention or control arms were reported separately. Effect size consisted of mean difference (MD) and 95% CI between the outcomes of intervention and the control groups using the generic inverse-variance random effects model. A two-sided *p*-value 0.05 was considered statistically significant. Heterogeneity between trial results was tested using the *I*^2^ statistic [16] and a value over 50% indicated a significant level of heterogeneity. Stability of results and possible source of heterogeneity between the studies was explored through sensitivity analyses by excluding results of one study at the time. In the analysis where SDs changes form baseline were imputed, a sensitivity analysis using also a 0.25 and a 0.75 correlation coefficients were performed to confirm the results. Moreover, subgroup analyses were used to evaluate the influence of some factors, including study design, geographical area, length of the study, and weight status. Publication bias was examined by visual inspection of the funnel plots. The analyses were carried out using Review Manager version 5.2 (RevMan 5.2, The Nordic Cochrane Centre, The Cochrane Collaboration, Copenhagen, Denmark).

## 3. Results

### 3.1. Study Selection and Main Characteristics 

**Figure 1:**
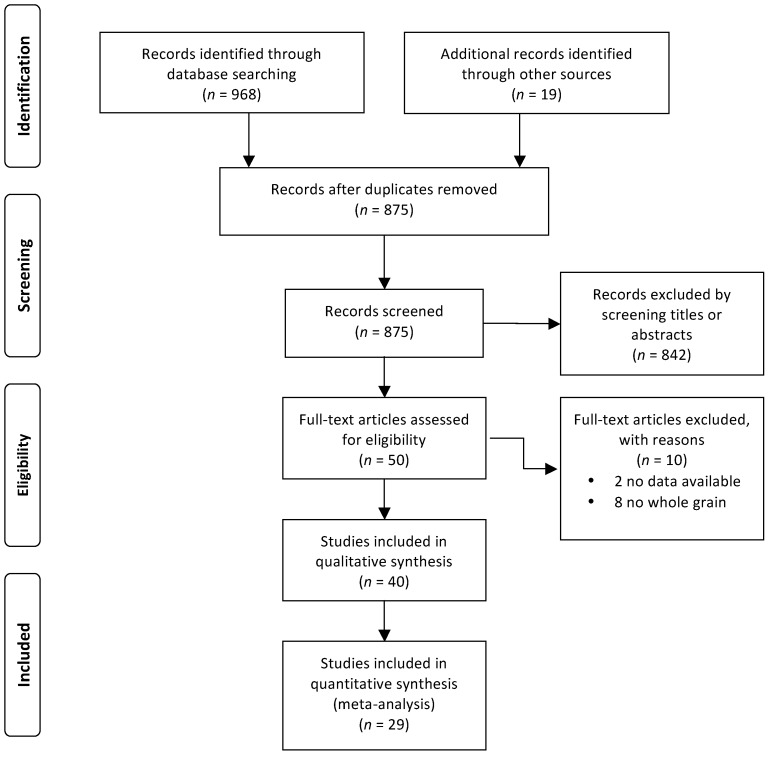
PRISMA flowchart indicating the results of the search strategy.

The flow chart of the search strategy is showed in Figure 1. From the initial 875 studies, a total of 51 articles were considered for the full-text examination. After the exclusion of 10 studies, 41 articles met the inclusion criteria for the qualitative analysis, while 30 were included in the quantitative meta-analysis.

Table 1 shows the characteristics of the 41 RCTs, which included 1033 healthy subjects (587 males and 682 females). Trials were conducted across 10 countries, including Sweden (eight trials) [17,18,19,20,21,22,23,24], Finland (five trial) [25,26,27,28,29], Canada (five trials) [30,31,32,33,34], UK (five trials) [4,35,36,37,38], Australia (three trial) [39,40,41], Denmark (three trials) [42,43,44], Italy (three trials) [5,45,46], USA (three trials) [47,48,49], Germany (one trial) [50], Japan (one trial) [51], Kuwait (one trial) [52], Singapore (one trial) [53], Spain (one trial) [54] and Switzerland (one trial) [55]. Five studies [35,37,42,45,47] had a parallel design and 36 [4,5,17,18,19,20,21,22,23,24,25,26,27,28,29,30,31,32,33,34,36,38,39,40,41,43,44,46,48,49,50,51,52,53,54,55] had a cross-over design. Twenty-five studies [17,18,19,20,21,22,24,25,26,27,28,29,30,31,32,33,34,40,41,43,44,46,49,52,53,54] evaluated the effects of the consumption of a single WG meal in an acute study, while 16 studies [4,5,23,32,35,36,37,38,39,40,41,42,45,47,48,50,51,55] evaluated the effects of a medium/long-term consumption in a RCT study with an intervention period ranging from two weeks to 16 weeks (median six weeks). The totality of the study included scored high quality. Even though studies were conducted on healthy individuals, 22 enrolled normal weight individuals [17,18,19,20,21,22,24,25,26,31,34,40,41,43,44,46,49,51,52,53,54,55], 11 overweight individuals [5,23,27,28,29,30,32,36,37,38,47], and seven obese individuals [33,35,39,42,45,48,50].

In the studies evaluating the acute effects of the consumption of a single WG meal, a wide variety of different meals were compared with control meals including mainly white wheat bread (Table 1). As regards medium/long-term RCT in 12 studies [5,23,35,36,37,38,39,40,41,48,50,51,55], participants consumed a diet in which food with refined cereals were substituted with WG foods, while in four studies the intervention groups were invited to consume WG sourdough bread [32], WG biscuits [45], WG breakfast cereals [4] or WG oats [47] during the normal diet. 

### 3.2. Acute Studies

#### 3.2.1. Glucose iAUC

Fourteen studies reported data on glucose iAUC, two of which relative to the time period 0–90 min [24,52], 10 to the time period 0–120 min (145 subjects) [18,19,20,21,31,34,40,46,53,54], four to the time period 0–180 min (61 subjects) [25,27,43,49], and two to the time period 0–240 [28,41]. Four studies were not reported because there was no information on the AUC calculation or because of different type of AUC was used [17,29,33,44]. Six studies [24,28,34,41,44] did not present relevant data for the meta-analysis. 

**Figure 2:**
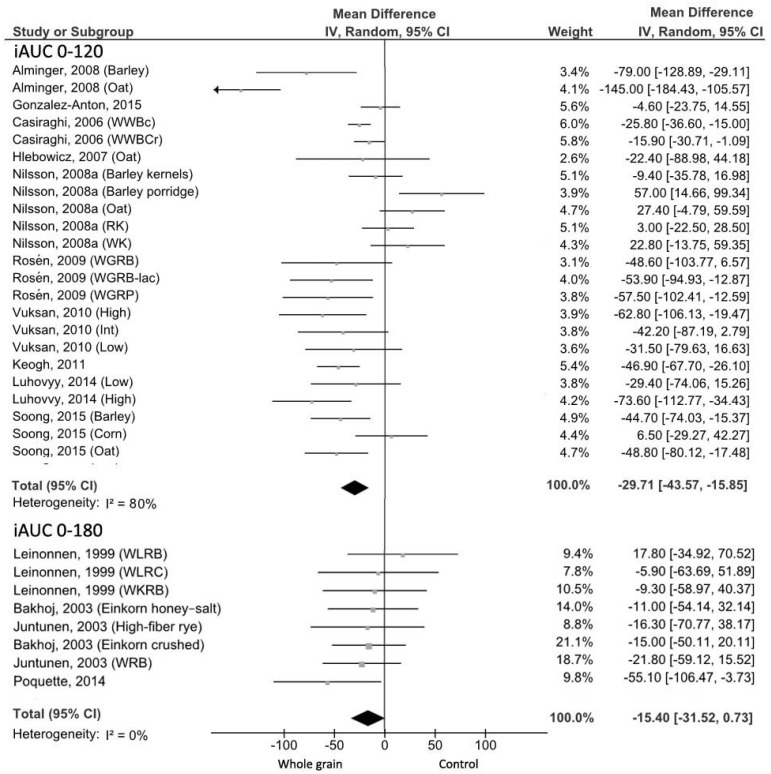
Forest plot of the meta-analysis carried out to investigate the effect of whole grain consumption on glucose iAUC. iAUC: incremental area under the curve; WWBc, whole-wheat cookies; WWBCr, whole-wheat crackers RK, rye kernels; WK, wholegrain wheat kernels; WGRB, whole grain rye bread; WGRB-lac, whole grain rye bread with lactic acid; WGRP, whole grain rye porridge; WLRB, wholemeal rye bread; WLRC, wholemeal rye crispbread; WKRB, whole kernel rye bread; WRB, Whole rye bread.

Plasma glucose concentrations after the WG meal for the time periods 0–120 min were lower compared with the control meal (MD = −29.71 mmol min/L, 95% CI: −43.57, −15.85 mmol min/L; Figure 2). Significant heterogeneity was found between studies (*I*^2^ = 80%; *p* <0.001). 

The visual inspection of the funnel plot showed asymmetry due to the study of Alminger et al. [18] (Supplementary Materials, Figure S1). Heterogeneity was reduced to 40% (*p* = 0.06) when three studies were excluded [18,20,54], with no change in the results (MD = −36.46 mmol min/L, 95% CI: −46.80, −26.12 mmol min/L), despite no substantial differences between these three studies and the others have been observed. These results did not achieve statistical significance in the 0–180 min analysis (MD = −15.40 mmol min/L, 95% CI: −31.52, 0.73 mmol min/L; Figure 2) with no evidence of heterogeneity (*I*^2^ = 0%, *p* = 0.76). 

Among the excluded studies, glucose response after consumption of the WG meal did not significantly differ from the refined grain meal across all the studies (Table 1). 

#### 3.2.2. Insulin iAUC

Insulin iAUC 0–120 min was evaluated in 5 studies [17,18,21,40,54] (58 subjects), while 8 studies [25,27,29,30,33,43,46,49] evaluated the iAUC 0–180 min (83 subjects). Two studies reported data on insulin iAUC relative to the time period 0–240 min [28,41] and were excluded from the analysis. Three studies were excluded because there was no information on the AUC calculation or because of different type of AUC was used [17,29,33]. 

**Figure 3:**
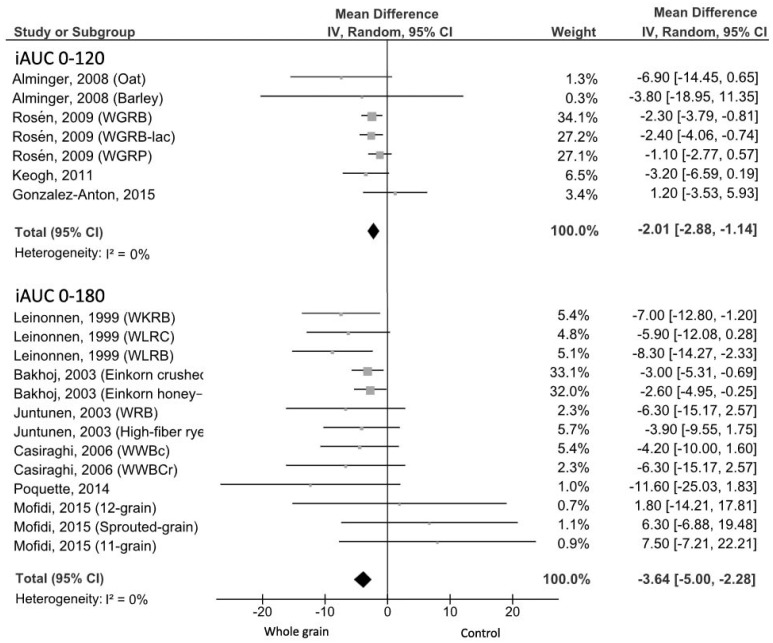
Forest plot of the meta-analysis carried out to investigate the effect of whole grain consumption on insulin iAUC. iAUC: incremental area under the curve; WGRB, whole grain rye bread; WGRB-lac, whole grain rye bread with lactic acid; WGRP, whole grain rye porridge; WKRB, whole kernel rye bread; WLRB, wholemeal rye bread; WLRC, wholemeal rye crispbread; WRB, Whole rye bread; WWBc, whole-wheat cookies; WWBCr, whole-wheat crackers.

The WG products induced significantly lower 0–120 min incremental areas than foods with lower amount or no WG foods (MD = −2.01 nmol min/L, 95% CI: −2.88, −1.14 nmol min/L; Figure 3), with no evidence of heterogeneity (*I*^2^ = 0%; *p* = 0.49). In the studies evaluating the iAUC 0–180 min, a significant insulin iAUC reduction with no evidence of heterogeneity was found (MD = −3.64 nmol min/L, 95% CI: −5.00, −2.28 nmol min/L; *I*^2^ = 1%, *p* = 0.44; Figure 3). Four [17,28,29,41] out of the five excluded trials showed a significantly smaller iAUC for the WG consumption in comparison with the WWB, while one study [33] found no difference between the WG and refined grain meals.

#### 3.2.3. Maximal Glucose and Insulin Response 

**Figure 4:**
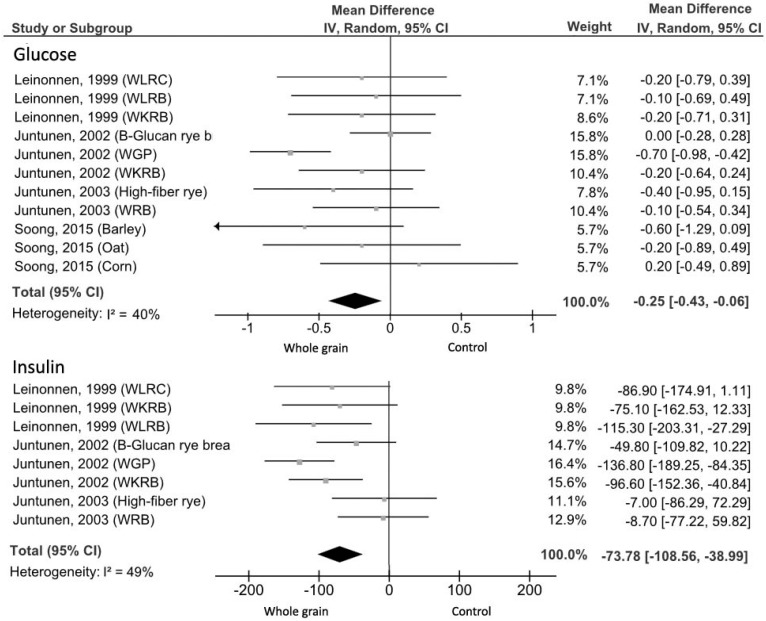
Forest plot of the meta-analysis carried out to investigate the effect of whole grain consumption on maximal glucose and insulin response. WLRC, wholemeal rye crispbread; WLRB, wholemeal rye bread; WKRB, whole kernel rye bread; WGP, whole grain pasta; WKRB, whole kernel rye bread; WRB, Whole rye bread.

Four studies [25,26,27,53] for a total of 71 subjects and 11 different test meals considered maximal glucose and insulin response incremental as outcome. The meta-analysis showed a significantly lower maximal glucose response (MD = −0.25 mmol/L, 95% CI: −0.43, −0.06 mmol/L) with low evidence of heterogeneity (*I*^2^ = 40%; *p* = 0.08) for the experimental compared with control groups (Figure 4). Concerning the study of Zafar et al. [52], blood glucose concentrations were significantly lower after WG products compared to refined food, however it was excluded from the analysis due to the lack of data. As regards maximal insulin responses, all test meals products induced a lower maximal insulin response with a MD of −73.78 pmol/L (95% CI: −108.56, −38.99 pmol/L) and moderate evidence of heterogeneity (*I*^2^ = 49%; *p* = 0.05; Figure 4).

### 3.3. Medium- and Long-Term Studies

#### 3.3.1. Fasting Glucose

**Figure 5:**
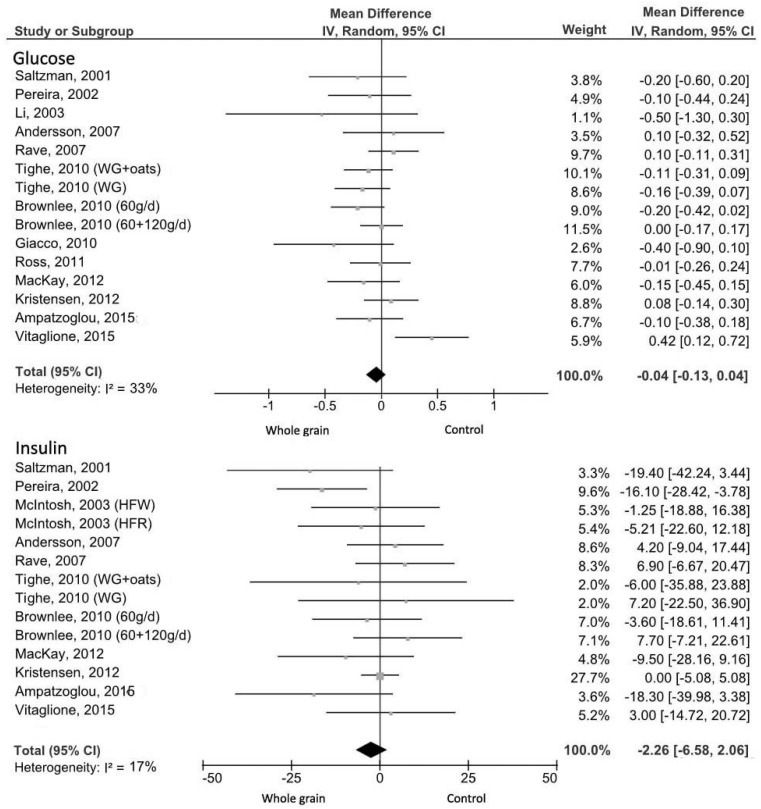
Forest plot of the meta-analysis carried out to investigate the effect of whole grain consumption on fasting glucose and insulin. WG, whole grain; HFW, high-fibre wheat; HFR, high-fibre rye.

In total, 10 crossover studies [4,5,23,32,36,39,48,50,51,55] and five parallel studies [35,37,42,45,47] accounting for 773 subjects (325 males and 448 females) reported fasting glucose outcomes for medium- and long-term WG meals intake. Two studies [4,39] were excluded because baseline fasting glucose values were not reported. When the results from all of the studies were grouped in a meta-analysis, there was no summary effect on fasting glucose in the WG group compared to the control group (MD = −0.04 mmol/L, 95% CI: −0.13, 0.04 mmol/L) with no significant evidence of heterogeneity (*I*^2^ = 33%; *p* = 0.11; Figure 5). The visual inspection of the funnel plot showed asymmetry due to the study of Li et al. (Supplementary Material, Figure S2) [51]. Sensitivity analyses showed that, after excluding results of Vitaglione et al. [45], where subjects were selected specifically with unhealthy dietary and lifestyle behaviours, and Rave et al. [50], where participants were obese subjects with elevated fasting blood glucose, summary effect size resulted in a low significant reduction of fasting glucose in favour of the intervention group with no evidence of heterogeneity (MD = −0.08 mmol/L, 95% CI: −0.16, −0.01 mmol/L; *I*^2^ = 0%, *p* = 0.72).

#### 3.3.2. Fasting Insulin

Plasma insulin was evaluated in 13 studies [4,5,23,32,35,37,38,39,42,45,47,48,50] with a total of 730 subjects (355 males and 375 females) and the meta-analysis was not significantly different in the WG group compared to the controls (MD= −2.26 pmol/L, 95% CI: −6.58, 2.06 pmol/L; *I*^2^ = 17%, *p* = 0.27; Figure 5). Two studies [4,5] were excluded for the lack of baseline values.

#### 3.3.3. HOMA-IR

**Figure 6:**
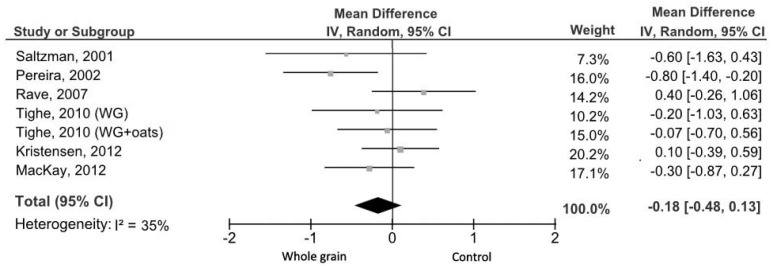
Forest plot of the meta-analysis carried out to investigate the effect of whole grain consumption on HOMA-IR. WG, whole grain.

Data on HOMA-IR were reported in seven studies [5,32,37,42,47,48,50] accounting for 377 healthy subjects (152 males and 225 females). The study of Giacco et al. [5] was excluded because of the lack of baseline values. The results of the meta-analysis showed no evidence of an effect on HOMA-IR for the medium- and long-term WG consumption compared to the control group (MD = −0.18, 95% CI: −0.48, 0.13) with no significant evidence of heterogeneity (*I*^2^ = 35%; *p* = 0.16; Figure 6). Sensitivity analyses showed that the exclusion of two studies [42,50] changed the summary effect size from non-significant to significant (MD = −0.39, 95% CI: −0.69, −0.08; *I*^2^ = 0%, *p* = 0.52). A possible reason for heterogeneity relied on the fact that the intervention, in both studies, was conducted in the context of a strict hypocaloric diet.

#### 3.3.4. Subgroup Analyses

Table 2 shows the results of categorical subgroup analyses for the effect of medium- and long-term consumption of wholegrain meals on fasting glucose and insulin. The subgroup analyses of fasting glucose concentrations indicated that the overall outcome of fasting glucose did not differ between subgroups. Instead, a significant reduction in fasting insulin concentrations were observed for studies not conducted in the European region (MD = −10.71 pmol/L, 95% CI: −18.19, −3.24 pmol/L). No significant heterogeneity was observed across the studies (*I*^2^ = 0%). The sensitivity analysis of the duration subgroups resulted in a significant lower fasting insulin (MD = −8.17 pmol/L, 95% CI: −15.24, −1.10 pmol/L; *I*^2^ = 19%, *p* = 0.28) after the removing of the study of Rave et al. [50], which was conducted on obese subjects with elevated fasting blood glucose. 

## 4. Discussion

The present study showed significant advantages in iAUC peak and incremental, both for post-prandial glucose and insulin in favour of the WG compared to control meals in acute studies. In some studies, benefits on glycaemic excursions [20,56], feeling of fullness, lower feeling of hunger and lower desire to eat [22] were found after the consumption of a WG meal. Overall, these results contribute to explain, from a mechanistic point of view, the association between WG food consumption and lower risk of type-2 diabetes [57], metabolic syndrome [58], and CVD [59] in epidemiological studies. Diets rich in refined carbohydrates induce a rapid increase in blood glucose concentrations with a high demand for insulin from the pancreatic β-cells [60], which in turn may increase the risk of insulin resistance [61]. In contrast, WG cereals are able to acutely lower blood glucose levels, which may improve insulin sensitivity and beta-cell function.

In relation to the postprandial blood glucose response, the difference between WG and refined grain meals was more relevant and statistically significant when the two hours’ response was evaluated. This is in line with other studies on the impact of different carbohydrates foods on postprandial blood glucose [62,63,64]. In fact, meal carbohydrates influence directly the early postprandial blood glucose response, whereas the late blood glucose levels are linked mainly to the hormonal (mainly insulin and glucagon) and metabolic (free fatty acids) response. 

Results from medium and long-term trials showed no statistically significant difference in HOMA-IR, fasting plasma glucose and insulin levels after WG compared to refined grain meals. This is not in line with evidence reported in some cross-sectional studies [62,64]. However, after a sensitivity analysis with exclusion of studies including obese individuals with unhealthy dietary habits at baseline, both fasting plasma glucose and HOMA-IR showed a significant difference between WG and the control meal. This suggests that the evidence available from intervention trials on the long-term effects of WG is still inadequate to drawn definitive conclusions and the analysis on a more homogenous group of individuals might lead to more consistent results.

The possible mechanisms for the beneficial effects of WG foods include the slow rate of digestion and the fermentation of fibre and resistant starch by microbiota in the large intestine with the production of short-chain fatty acids (SCFA). SCFA in the liver increase glucose oxidation, decrease fatty acid release, and increase insulin clearance, thus improving glucose homeostasis and insulin sensitivity [65]. Moreover, the pancreatic beta cells are extremely sensible to oxidative damage [66] and could be protected by some antioxidants present in WG cereals, such as polyphenols (ferulic acid, lignans, and anthocyanins) and alkylresorcinols, demonstrated to be direct radical scavengers in animal models [67]. Nevertheless, the ability of WG cereals to improve glycaemic control may be related to the synergistic action of multiple compounds more than a single component [68].

The results of this meta-analysis should be considered in light of some limitations. First, the lack of a clear universal definition of the concept of WG food as well as the different properties of the source of dietary WG could lead to some bias. Separate investigations across the various whole grain foods produced from different crops may be useful to reduce heterogeneity and to obtain a more robust scientific evidence of the impact of WG from specific sources on glycaemic responses. Second, the sample sizes of some RCTs were relatively small and some of the variables analysed were not primary outcomes of the studies. Third, the imputation of SD could lead to a bias of the results. However, the sensitivity analyses imputing different values of correlation confirmed that the general results were robust. Fourth, several studies were not included due to the lack of available information necessary for the quantitative analysis; as some of these studies reported not significant results, the possibility of selection bias should be considered. Finally, only few studies reported HbA1c and no conclusions about the effect of WG foods on blood glucose levels over a period of time can be made.

In conclusion, the results of the present meta-analysis suggest that consumption of WG foods may improve acutely the postprandial glucose and insulin homeostasis compared to similar refined foods in healthy subjects. These effects, in addition to a better appetite regulation, need to be confirmed by future long-term studies designed ad hoc to test whether WG foods may contribute to reduce the risk of type 2 diabetes and other chronic diseases. Moreover, the results of the acute studies included in this meta-analysis relied mostly on studies evaluating the effects of oat, rye and barley; these cereals provide a relatively lower contribution to the overall cereal intake worldwide [69] than wheat. In fact, WG wheat products have a larger diffusion at the population level, at least in Western countries. Therefore, further research is needed to better understand the long-term effects and the biological mechanisms that underline the health benefits of WG food consumption.

## Figures and Tables

**Table 1:** Characteristics and main findings of the clinical trials evaluating the effects of WG consumption in healthy subjects.

Author, Year (Reference)	Country	Design (Washout or Arms)	Participants, Age, Year	BMI, kg/m^2^	Test Meals	Matching	Duration	Outcomes Evaluated	Main Results	Study Quality
**Acute Effect**										
Stefoska-Needham, 2016	Australia	C (3d)	40 (20M/20F), 29.3	23.4	I: Whole sorghum biscuits C: Wheat biscuits	none	-	Glucose and insulin iAUC	Greater insulin response (iAUC 4 h) after the red sorghum biscuit	No data
Gonzalez-Anton, 2015	Spain	C (1w)	23 (13M/10F), 25 ± 1	23.3 ± 0.5	I: Wholemeal C: WWB	50 g of available carbohydrates	-	Glucose and insulin iAUC	There were no differences in glucose and insulin iAUC	
Johansson, 2015	Sweden	C (6d)	23 (7M/16F), 60.1 ± 12.1	23.8 ± 3.4	I: uRCB I2: RCB C: WCB	none	-	Glucose and insulin iAUC	Insulin response was lower for RCB (10%) and uRCB (21%) compared with WCB	TOTAL AUC
Mofidi, 2015	Canada	C (1w)	12M, 54.9 ± 2.0	29.1 ± 1.1	I1: 11-grain I2: Sprouted-grain I3: 12-grain C: WBB	50 g of available carbohydrates	-	Insulin iAUC	Only sprouted-grain improved postprandial glucose and insulin response	
Soong, 2015	Singapore	C (na)	12 (4M/8F), 26.2 ± 5.3	20.2 ± 1.7	I: WG barley flour I2: WG oat flour I3: WG yellow corn flour C: Refined wheat flour	50 g of available carbohydrates	-	Glucose peak and iAUC	Improved postprandial glucose response for I1 and I2 but not for I3	
Zafar, 2015	Kuwait	C (na)	13F, 21.4 ± 2.3	23.6 ± 2.4	I: WGB C: WWB	25 g available carbohydrate	-	Glucose peak and iAUC	Lower glucose peak and iAUC	
Luhovyy, 2014	Canada	C (na)	30M, 22.9 ± 0.6	22.6 ± 0.3	I1: WG maize (high) I2: WG maize (low) C: Cookies	none	-	Glucose iAUC	Reduction in postprandial glucose response	
Moazzami, 2014	Finland	C (1–2w)	20F, 61.0 ± 4.8	26.0 ± 2.5	I: WRB C: WWB	50 g of available carbohydrates	-	Glucose and insulin iAUC	Improved postprandial insulin response but not glucose response	15
Poquette, 2014	USA	C (1w)	10M, 25.1 ± 4.0	24.2 ± 2.8	I: Sorghum flour C: Wheat flour	50 g of total starch	-	Glucose and insulin iAUC	Improved postprandial glucose and insulin response	15
Lappi, 2013	Finland	C (3d)	15 (6M/9F), 57	26	I: WRB C: WWB	50 g of available carbohydrates	-	Glucose and insulin iAUC	Improved postprandial insulin response but not glucose response	15
Keogh, 2011	Australia	C (2d)	10F, 29.4	21.8	I: WGB C: WWB	none	-	Glucose and insulin iAUC	Improved postprandial glucose and insulin response	
Vuksan, 2010	Canada	C (2d)	11 (6M/5F), 30 ± 3.6	22.3 ± 2.8	I1: WG low I2: WG intermediate I3: WG high C: WWB	50 g of available carbohydrates	-	Glucose iAUC	Reduction in postprandial glucose response	
Rosén, 2011	Sweden	C (1w)	10 (5M/5F), 26.0 ± 1.1	22.6 ± 0.4	I1: WGRB I2: WGRB-lac I3: RK I4: WK C: WWB	50 g of available carbohydrates	-	Glucose and insulin total AUC, incremental glucose and insulin peak	Lower early glucose responses (0–60 min), insulin response and incremental glucose and insulin peak	15
Kristensen, 2010	Denmark	C (na)	16 (6M/10F), 24.1 ± 3.8	21.7 ± 2.2	I1: WGB I2: WGP C: WWB and pasta	50 g of available carbohydrates	-	Glucose iAUC	No differences between any WG product and R product	15
Hlebowicz, 2009	Sweden	C (1w)	10 (3M/7F), 26 ± 1	24.1 ± 0.8	I: WRB C: WWB	50 g of available carbohydrates	-	Glucose iAUC	No differences between WG product and R product	15
Najjar, 2009	Canada	C (>1w)	10M, 59 ± 2.41	30.8 ± 0.95	I1: WGB I2: WG barley C: WWB	50 g of available carbohydrates	-	Glucose and insulin AUC	No differences between WG product and R product	15
Rosén, 2009	Sweden	C (1w)	12 (9M/3F), 25.3 ± 0.8	23.1 ± 0.6	I1: WGRB I2: WGRB-lac I3: WGRP C: WWB	40 g of available carbohydrates	-	Glucose and insulin iAUC	Improved postprandial glucose and insulin response for WG products	15
Alminger, 2008	Sweden	C (1w)	13 (9F/4M), 56 ± 13.2	24.4 ± 2.6	I1: Oat I2: Barley C: Glucose load	25 g of available carbohydrates	-	Glucose and insulin iAUC	Improved postprandial glucose and insulin response for WG products	15
Nilsson, 2008	Sweden	C (>3d)	12 (7M/5F), 28.3 ± 5.1	22.1 ± 2.0	I1: WK I2: RK I3: Oat kernels I4: Barley kernels I5: WG barley porridge C: WWB	50 g of available carbohydrates	-	Glucose iAUC	Improved postprandial glucose response for RK and barley kernels consumption	15
Hlebowicz, 2007	Sweden	C (>1w)	12 (6M/6F), 28 ± 4	22 ± 2	I1: WG oat flakes C: Cornflakes	None	-	Glucose iAUC	No differences between WG product and R product	15
Casiraghi, 2006	Italy	C (2w)	10 (5M/5F), 25.4 ± 0.5	22.6 ± 0.7	I1: WWBCr I2: WWBc I3: BCr I4: BC C: WWB	40 g of available carbohydrates	-	Glucose and insulin iAUC	Improved postprandial glucose and insulin response for WG products	15
Bakhoj, 2003	Denmark	C (1w)	11M, 25 ± 2	23 ± 4	I1: Einkorn honey–salt I2: Einkorn crushed C: Wheat	50 g of available carbohydrates	-	Glucose and insulin total AUC	No differences between WG product and R product	15
Juntunen, 2003	Finland	C (1–2w)	19F, 61 ± 4.8	26 ± 2.5	I1: WRB I2: High-fibre rye bread C: WWB	50 g of available carbohydrates	-	Glucose and insulin iAUC, maximal glucose and insulin response	Improved postprandial insulin response for WRB intake and maximal insulin response for both WRB and High-fibre rye bread. No differences for glucose iAUC and maximal response for any WG products	15
Juntunen, 2002	Finland	C (1–2w)	20 (10M/10F), 28.5 ± 1.8	22.9 ± 1	I1: WKRB I2: β-glucan rye bread I3: WGP C: WWB	50 g of available carbohydrates	-	Maximal glucose and insulin response	Improved maximal glucose response for WGP and improved maximal insulin response for all the WG meals	15
Leinonnen, 1999	Finland	C (na)	20 (10M/10F), M 32 ± 3 F 27 ± 5	M 24.5 ± 2.2; F 20.3 ± 1.1	I1: WKRB I2: WRB I3: WRC C: WWB	50 g of available carbohydrates	-	Glucose and insulin iAUC, maximal glucose and insulin response	Improved insulin iAUC and maximal response for WKRB intake. No differences for glucose iAUC and maximal response for any WG products	15
Medium-long term effect										
Ampatzoglou, 2015a	UK	C (4w)	33 (12M/21F), 48.8 ± 1.1	27.9 ± 0.7	I: WG pasta, rice, snacks, breakfast cereals C: RG pasta, rice, snacks, breakfast cereals	isoenergetic (2000 kcal/day)	6w	Fasting glucose	Fasting glucose did not differ between groups	14
Ampatzoglou, 2015b	UK	C (4w)	33 (12M/21F), 48.8 ± 1.1	27.9 ± 0.7	I: WG pasta, rice, snacks, breakfast cereals C: RG pasta, rice, snacks, breakfast cereals	isoenergetic (2000 kcal/day)	6w	Fasting insulin	Fasting insulin did not differ between groups	14
Vitaglione, 2015	Italy	P (2 arms)	68 (23M/45F), I: 40 ± 2 C: 37 ± 2	I: 30.0 ± 0.5 C: 29.5 ± 0.4	I: 3 WG biscuits C: 1 package of crackers and 3 slices of toasted bread	isoenergetic (1500 kcal/day)	8w	Fasting glucose and insulin	Fasting glucose and insulin did not differ between groups	13
Kristensen, 2012	Denmark	P (2 arms)	72F, I: 60.3 ± 5.3 C: 59.1 ± 5.6	I: 30.4 ± 0.6 C: 30.0 ± 0.4	I: WG pasta, bread and biscuits C: RG pasta, bread and biscuits	hypocaloric (300–1200 kcal/day)	12w	Fasting glucose and insulin, HOMA and HbA1c	HbA1c, fasting glucose and insulin, and HOMA did not differ between groups	14
MacKay, 2012	Canada	C (4–5w)	14 (10M/4F), 53 ± 6.0	26.5 ± 2.9	I: WG sourdough bread C: WWB	isoenergetic	6w	Fasting glucose and insulin and HOMA	Fasting glucose and insulin and HOMA did not differ between groups	13
Ross, 2011	Switzerland	C (5–7w)	17 (6M/11F), M 36.5 ± 4.2 F 34.1 ± 3.0	M 24.5 ± 0.6 F 23.1 ± 0.8	I: WG pasta, rice, snacks and breakfast cereals C: RG pasta, rice, snacks and breakfast cereals	isoenergetic (2000 kcal/day)	2w	Fasting glucose	Fasting glucose did not differ between groups	13
Brownlee, 2010	UK	P (3 arms)	266 (132M/134F), 45.7 ± 10	I: 30.0 ± 3.7 C:30.3 ± 4.5	I: WG pasta, rice, snacks and breakfast cereals C: RG pasta, rice, snacks and breakfast cereals	none	16w	Fasting glucose and insulin	Fasting glucose did not differ between groups	14
Giacco, 2010	Italy	C (none)	15 (12M/3F), 54.5 ± 7.6	27.4 ± 3.0	I: WG bread, pasta, rusks and crackers C: RG bread, pasta, rusks and crackers	isoenergetic (2000 kcal/day)	3w	Fasting glucose and insulin and HOMA	Fasting glucose and insulin and HOMA did not differ between groups	14
Tighe, 2010	UK	P (3 arms)	206 (104M/102F), I1: 51.6 ± 0.8 I2: 52.1 ± 0.9 C: 51.8 ± 0.83	I1: 28.0 ± 0.5; I2: 27.0 ± 0.4 C: 28.0 ± 0.5	I1: WG bread and WG cereals I2: WG wheat food plus oat C: RG bread and cereals	isoenergetic (2100 kcal/day)	16w	Fasting glucose and insulin and HOMA	Fasting glucose and insulin and HOMA did not differ between groups	13
Costabile, 2008	UK	C (2w)	31 (15M/16F), 25	20–30 range	I: WG cereals C: Wheat bran cereals	48 g/day portion	3w	Fasting glucose and insulin	No significant differences were observed	
Anderson, 2007	Sweden	C (6–8w)	30 (8M/22F), 59 ± 5	28.3 ± 2.0 30.0 ± 4.0 C;	I1: WG bread, crispbread, muesli, pasta, pancakes, scones, pie, pizza C: RG bread, crispbread, muesli, pasta, pancakes, scones, pie, pizza	isoenergetic (2100 kcal/day)	6w	Fasting glucose and insulin	Fasting glucose and insulin did not differ between groups	12
Rave, 2007	Germany	C (2w)	31 (13M/18F), 51 ± 13	33.9 ± 2.3	I: WG C: MR	isoenergetic (1700 kcal/day)	4w	Fasting glucose and insulin, HOMA	HOMA, fasting glucose and insulin did not differ between groups	
Li, 2003	Japan	C (4w)	10F, 20.4 ± 1.3	19.2 ± 2.0	I: Barley diet C: standard diet	isoenergetic (1900 kcal/day)	4w	Fasting glucose and HbA1	Fasting glucose and HbA1 did not differ between group	15
McIntosh, 2003	Australia	C (none)	28M, range 40-65	30 ± 0.9	I1: WG bread, crispbread and breakfast cereal I2: WG rye bread, rye crispbread and rye breakfast cereal C: WWB, RG crispbread and rice cereal	isoenergetic (2300 Kcal/day)	4w	Fasting glucose and insulin	Fasting glucose and insulin were lower in the WG groups	13
Pereira, 2002	USA	C (6–9w)	11 (5M-6F), 41.6 ± 2.67	30.2 ± 1.01	I: WG pasta, rice, snacks, breakfast cereals C: RG pasta, rice, snacks, breakfast cereals	isoenergetic (2000 kcal/day)	6w	Fasting glucose and insulin and HOMA	Fasting insulin and HOMA were significantly lower in the WG group	13
Saltzman, 2001	USA	P (2 arms)	43 (20M/23F), I: 45.1 ± 22.7 C: 44.1 ± 21.3	I: 26.1 ± 3.4 C: 26.7 ± 3.2	I: Standard diet plus WG oat C: Standard diet	hypocaloric (1900 kcal/day)	6w	Fasting glucose and insulin and HOMA	Fasting glucose and insulin and HOMA did not differ between groups	15

BC, barley cookies; BCr, barley crackers; C, control; HOMA, homeostatic model assessment; I, intervention; iAUC: incremental area under the curve; na, not available; RCB, fermented whole grain rye crisp bread; RG, refined grain; RK, rye kernels; uRCB, Unfermented whole grain rye crisp bread; WCB, refined wheat crisp bread; WG, Whole grain; WGB, whole grain bread; WGRB, whole grain rye bread; WGRB-lac, whole grain rye bread with lactic acid; WGRP, whole grain rye porridge; WGP, whole grain pasta; WK, wholegrain wheat kernels; WKRB, whole kernel rye bread; WRB, Whole rye bread; WRC, Whole rye Crispbread; WWB: white wheat bread; WWBc, whole-wheat cookies ; WWBCr, whole-wheat crackers.

**Table 2:** Subgroup analyses of medium- and long-term wholegrain intake effect on fasting glucose and insulin.

	Glucose				Insulin			
	No. of Datasets	OR (95% CI)	Heterogeneity		No. of Datasets	OR (95% CI)	Heterogeneity	
			*I*^2^ (%)	*p*			*I*^2^ (%)	*p*
Study design								
Parallel	8	−0.03 (−0.17, 0.11)	58	0.03	7	−0.14 (−4.40, 4.12)	0	0.61
Crossover	8	−0.04 (−0.15, 0.07)	0	0.49	6	−4.76 (−12.37, 2.86)	38	0.14
Geographical area								
Europe	11	−0.02 (−0.12, 0.08)	44	0.06	8	0.78 (−3.09, 4.66)	0	0.80
Other	4	−0.17 (−0.36, 0.02)	0	0.84	5	−10.71 (−18.19, −3.24)	0	0.59
BMI category								
Normal/overweight	9	−0.12 (−0.22, −0.02)	0	0.83	5	−3.15 (−12.14, 5.84)	2	0.40
Obese	5	0.04 (−0.11, 0.19)	58	0.04	8	−1.17 (−6.21, 3.86)	23	0.25
Duration								
≤6 weeks	10	−0.05 (−0.16, 0.05)	0	0.54	8	−5.93 (−13.31, 1.44)	13	0.38
>6 weeks	5	−0.01 (−0.16, 0.13)	64	0.02	6	0.56 (−3.78, 5.89)	0	0.89

## Supplementary Materials

The following are available online at www.mdpi.com/2072-6643/9/7/769/s1.
