# Supplementary material for: Whole Grain Intake and Glycaemic Control in Healthy Subjects: A Systematic Review and Meta-Analysis of Randomized Controlled Trials
Source: Nutrients. 2017 Jul 19;9(7):769. doi: 10.3390/nu9070769 (PMC5537883; doi:10.3390/nu9070769)
Supplement: Supplementary file 1 [file nutrients-09-00769-s001.zip › nutrients-200644-supplementary.pdf]

Supplementary Material

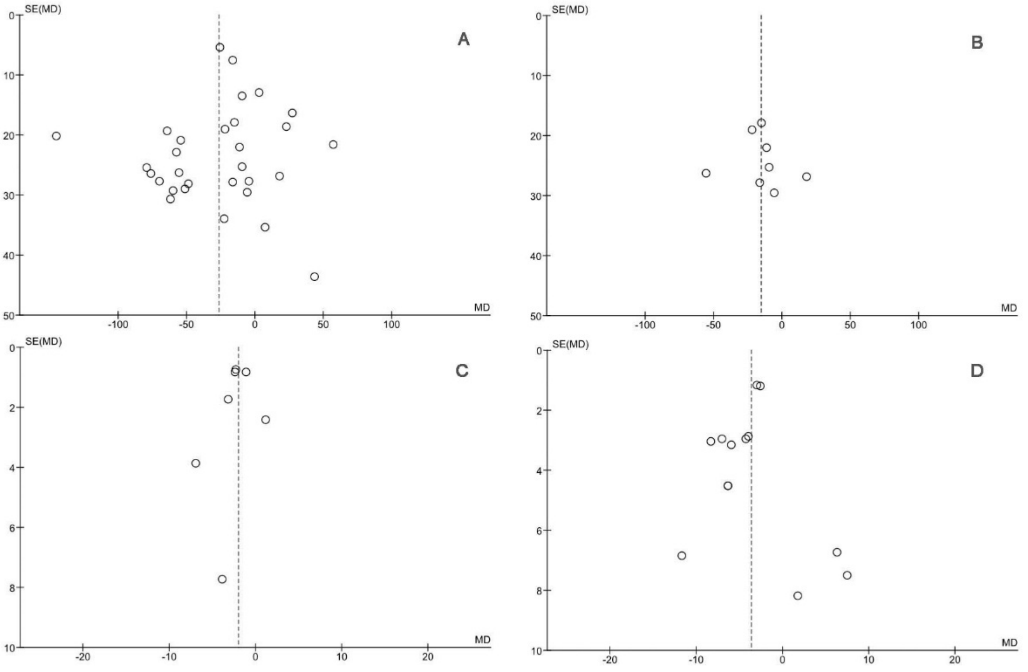

Supplementary Material Figure S1. Funnel plots for the effect of whole grain consumption on (**A**) Glucose iAUC 0–120; (**B**) Glucose iAUC 0–180; (**C**) Insulin iAUC 0–120 and (**D**) Insulin iAUC 0–180

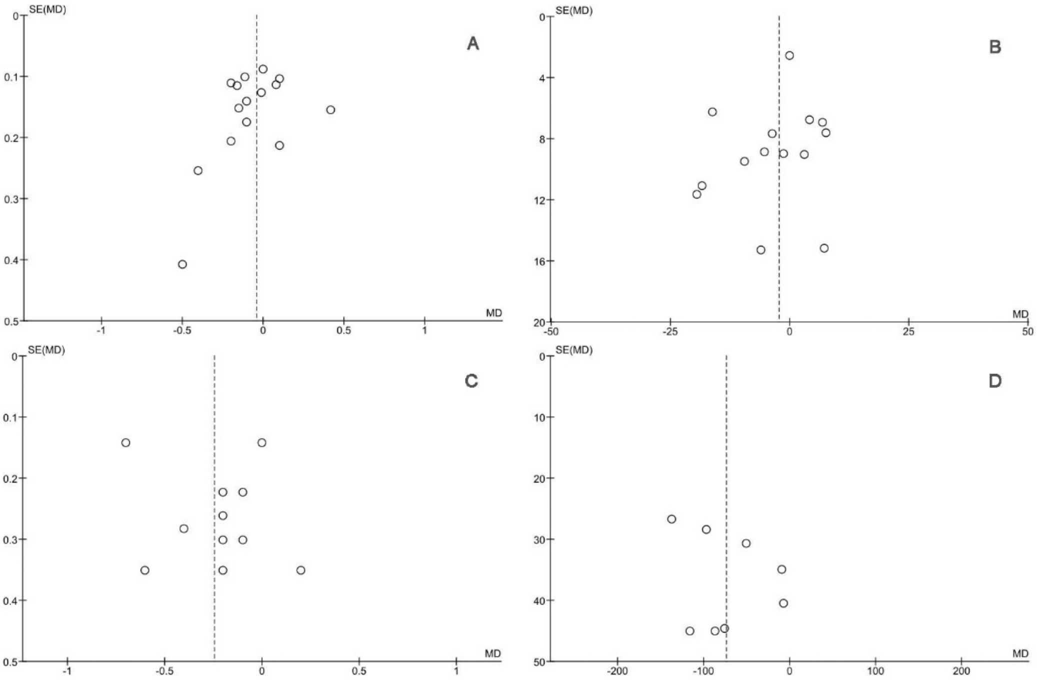

Supplementary Material Figure S2. Funnel plots for the effect of whole grain consumption on (**A**) Fasting glucose; (**B**) Fasting insulin; (**C**) Maximal glucose response and (**D**) maximal insulin response.
